# Expected benefit of genomic selection over forward selection in conifer breeding and deployment

**DOI:** 10.1371/journal.pone.0208232

**Published:** 2018-12-10

**Authors:** Yongjun Li, Heidi S. Dungey

**Affiliations:** 1 Scion (New Zealand Forest Research Institute), Rotorua, New Zealand; 2 Agriculture Victoria, AgriBio Centre, DEDJTR, Bundoora, Victoria, Australia; Aristotle University of Thessaloniki, GREECE

## Abstract

Genomic selection is a proven technology in animal and plant breeding to accelerate genetic gain, but as yet is to be fully realised in forest tree breeding. This paper examines, through stochastic simulation, the potential benefits of genomic selection (GS) over forward selection (FS) in a typical conifer breeding program. Methods of speeding the deployment of selected material were also considered, including top-grafting onto mature seed orchard ortets, using additional replicates of clones in archives for crossing, and embryogenesis and clonal propagation. Genetic gain per generation was found to increase considerably when the size of the training population was larger (800 c.f. 3000 clones), or when the heritability was higher (0.2 c.f. 0.5). The largest genetic gain, of 24% was achieved where large training populations (3000 clones) and high heritability traits (0.5) were combined. The accuracy of genomic breeding values (GEBVs) increased with the increase in the number of clones in the training population, the heritability of the trait and the density of the SNP markers. Calculated accuracies of simulated GEBVs and genetic gain per unit of time suggested that 2000 clones in the training population is the minimum size for effective genomic selection for conifers. Compared with forward selection, genomic selection with 2000 clones in the training population, and a 60K SNP panel, an increase of 1.58 mm per year in diameter-at-breast-height (DBH) and 2.44 kg/m^3^ per year for wood density can be expected. After one generation (9-years), this would be equivalent to 14.23 mm and 21.97 kg/m^3^ for DBH and wood density respectively. Deploying clones of the selected individuals always resulted in higher additional genetic gain than deploying progeny/seedlings. Deploying genetic material selected from genomic selection with top-grafting for early coning appeared to be the best option. Application of genomic selection to conifer breeding programs, combined with deployment tools such as top-grafting and embryogenesis are powerful tools to speed the delivery of genetic gain to the forest.

## Introduction

Many animal and plant breeders now use genomic selection routinely [1, 2, 3]. In forestry, genomic selection is yet to be fully realised. The benefits of genomic selection in forestry are potentially very large through shortening the long generation intervals, speeding the delivery of genetic gain, and reducing the reliance on field testing [4–6]. Benefits also include the estimation of both the additive genetic variance and the non-additive genetic variance [7], which potentially gives further benefits. Partitioning the non-additive genetic variance can add to the accuracy of breeding value estimates due to overall greater accuracy in the variance partitioning process. Trees also have the ability to be vegetatively propagated (cloned), so that non-additive genetic variance can be captured by deploying copies of outstanding genotypes [8, 9].

In practice, the accuracy of genomic selection directly depends on four factors [10, 11]: 1) the level of linkage disequilibrium between SNPs and quantitative trait loci (QTL), 2) the number of varieties/clones with phenotypes and genomic data in the training population from which the SNP effects are estimated, 3) the heritability of the trait in question, and 4) the distribution of QTL effects. The first two factors are under the control of the breeder and the other two are not [10]. Trait heritability can be improved through experimental design and testing strategies [12–15], although the underlying genetic control will remain the same. Linkage disequilibrium is a particularly important variable when considering the prospects of genomic selection in forest trees [4]. The level of linkage disequilibrium can be increased by reducing the effective population size and increasing SNP marker density [4]. A large effective population size leads to low level of linkage disequilibrium and, in turn, reduces the prediction accuracy in genomic selection [4, 16], however, it can also result in more recombination and a greater genetic diversity [17], which is favourable for long-term genetic gain [18].

Genomic tools have been used in research for a number of forestry species now [7, 19–28]. Despite this, some breeding programs are still reluctant to move even to forward selection from a backward selection strategy [29, 30]. Some practical simulations have been undertaken to determine the opportunity that cloning has for delivering genetic gain [9]. Very few simulations for the application of genomics in forestry have been published. Very few simulations have properly considered different deployment pathways that are available to practitioners in order to maximise the opportunity available through each of the different breeding pathways.

Clonal forestry is promising to provide quality planting material to forest growers in the shortest time. It is defined as the commercial production and deployment of plants of field-tested individual genotypes of forest tree species [31]. Clonal forestry is now a commercial reality for many conifer species, such as *Abies*, *Larix*, *Picea*, *Pinus*, and *Pseudotsuga* [32] and contributes approximately 5% of the total current New Zealand planting stock requirements. The advantages of clonal forestry in radiata pine include reducing the rotation age, and efficiently capturing additive and non-additive genetic gains [31, 33–35]. An ability to rapidly multiply valuable crosses is another key advantage [33]. The disadvantages include increases in operational cost [31] and reduction of genetic diversity [36, 37]. Clonal forestry for conifers is achieved mostly by the propagation of cuttings [38, 39] or by somatic embryogenesis [40]. Somatic embryogenesis is the most powerful way to deliver genetic gain to the forest because cell lines can be maintained in a juvenile state indefinitely through cryopreservation.

This paper will simulate the combination of breeding, genomics and deployment methods in order to critically examine the potential benefits of genomic selection in a typical conifer breeding program. Key variables tested include training population sizes, heritability and length of breeding cycles. Genetic gains will be estimated for combinations of variables for tree breeding and genomics approaches. Benefits from the different deployment options employed for the different breeding approaches will be important for the realisation of genetic gain [41]. We will simulate the genetic gain from deployment using embryogenesis from cryopreserved cell lines [40, 42, 43] and seed production, with the option of speeding seed production by topgrafting [44]. These simulations will, for the first time, clearly show the expected genetic gain per year for forest companies to strategically evaluate the opportunity that genomics offers for forest productivity.

## Materials and methods

### Assumptions and structures—Conifer breeding cycle timelines

**Fig 1:**
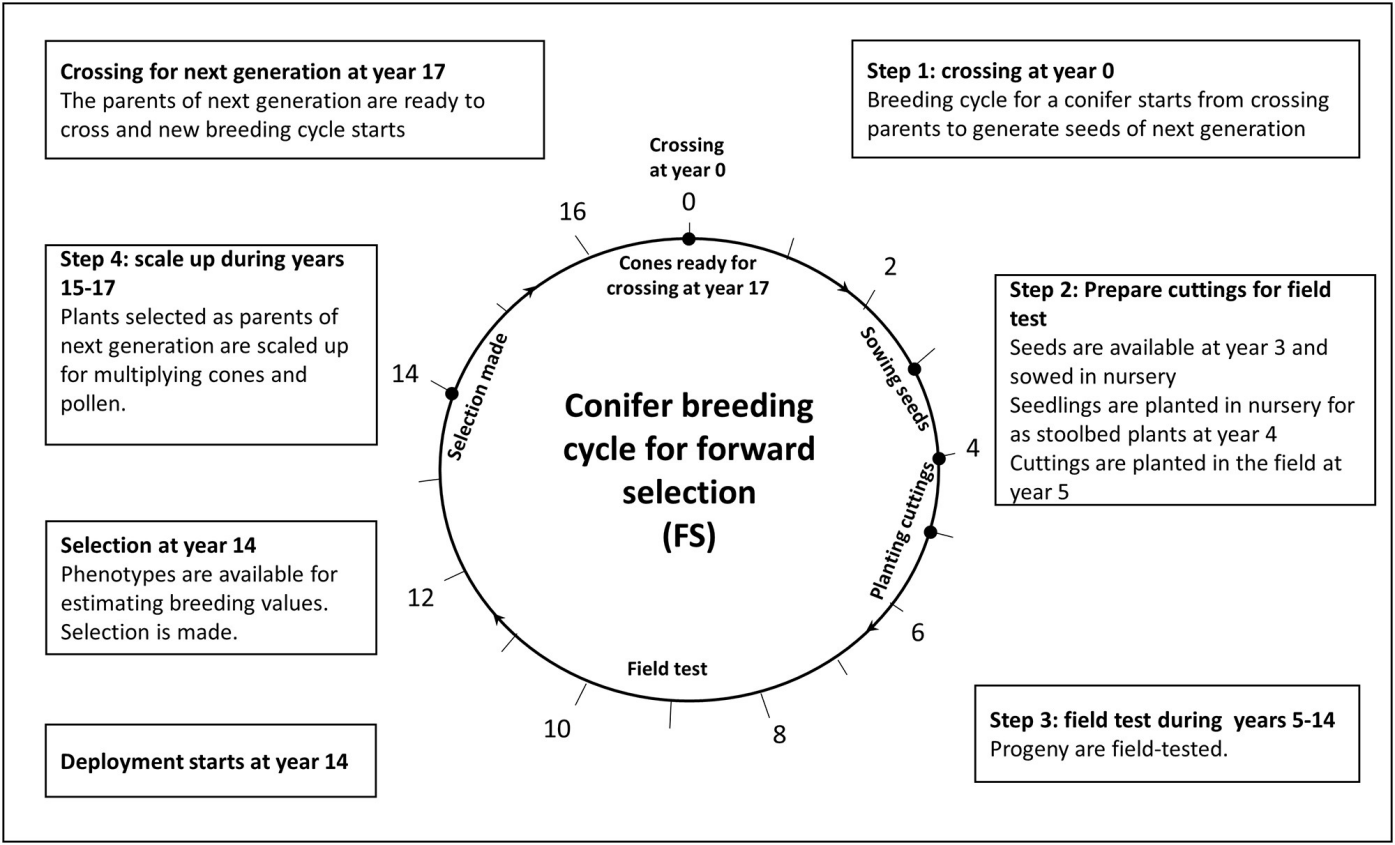
Example of conifer breeding cycle for conventional forward selection with a generation interval of 17 years.

**Fig 2:**
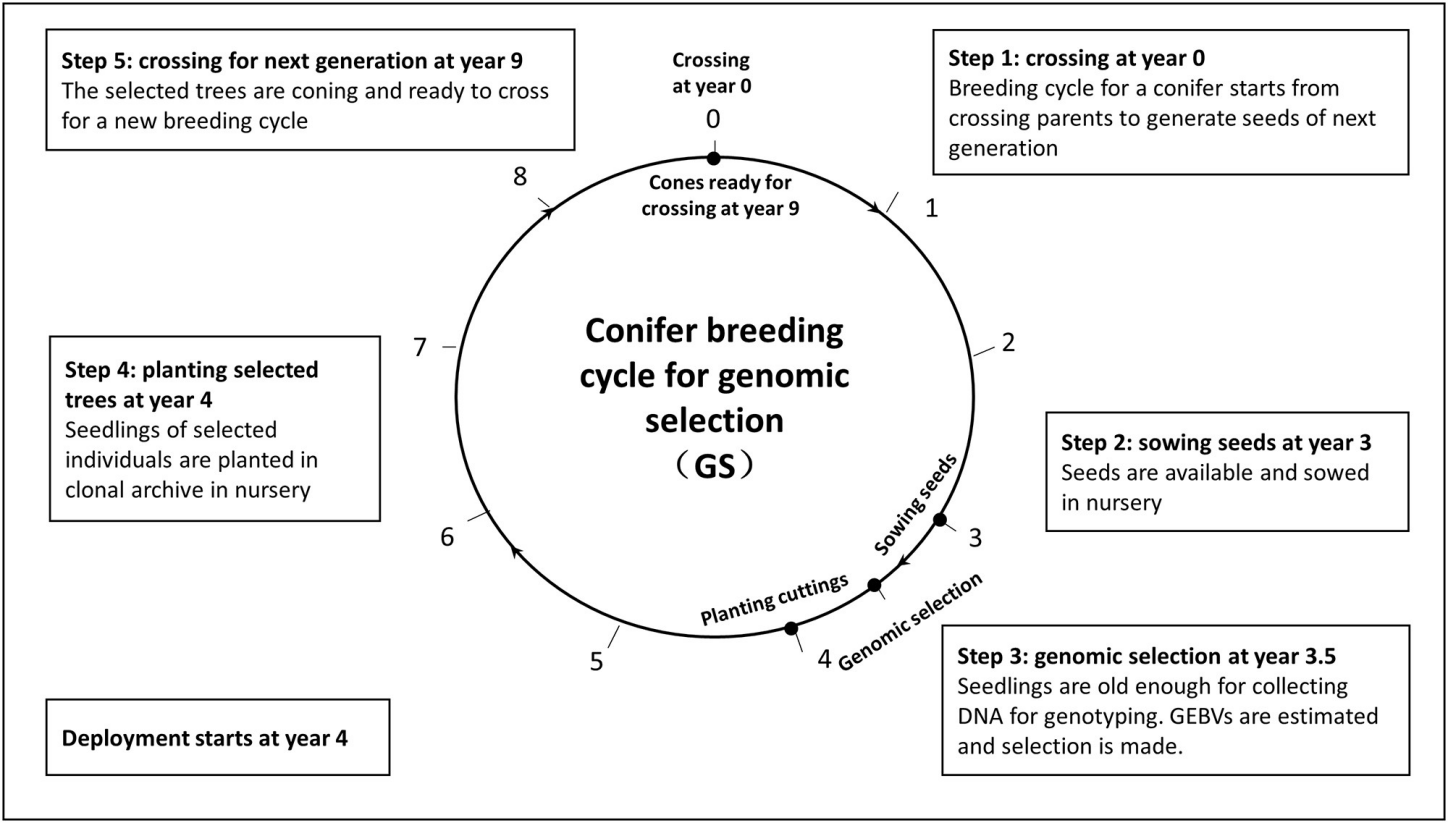
Example of conifer breeding cycle for genomic selection with a generation interval of 9 years.

We consider two primary conifer breeding approaches; forward selection with clonal testing (FS; Fig 1) and forward selection with genomics (GS, Fig 2). With FS, there are six steps per breeding cycle: 1) crossing parents and generating seeds in a crossing orchard (3 years), 2) sowing seed and preparing stool plants for cuttings (2 years), 3) progeny and field testing, with installation of a clonal crossing archive at the same time (9 years), and 4) scale up the selected individuals with sufficient cones for crossing (3 years). Therefore, the forward selection process has a generation interval of 17 years. Using a genomic selection strategy (GS), progeny field testing is not required. Selection candidates can be ranked based on genomic breeding values (GEBVs) that can be estimated when seedlings are only six months old. The selected trees are kept in a seed orchard for further 5.5 years before crossing can occur. Overall, generation interval can be reduced to 9 years with genomic selection (Fig 2).

One time-saving option for shorter breeding cycle relevant to the FS approach, is the establishment of a clonal archive (CA) at the time of progeny testing, where ramets of each clone under progeny testing are also kept in an archive for crossing on a site known to promote good coning. Clones are therefore available with cones for crossing right after selection is made. With this clonal archive option, step 4 is not needed (Fig 1) and the generation interval is reduced to 14 years. This option of forward selection is called forward selection with a clonal archive (FSCA). Top-grafting can also be used to graft juvenile scions of selected individuals onto the top of mature rootstock in order to accelerate coning [45, 46]. This top-grafting process is assumed to reduce the age of coning from 5 years to 3 years and, in turn, reduces the generation interval to 7 years. This option is relevant only to GS and is therefore called genomic selection with top grafting (GSTG).

### Assumptions and structures—Deployment pathways

**Fig 3:**
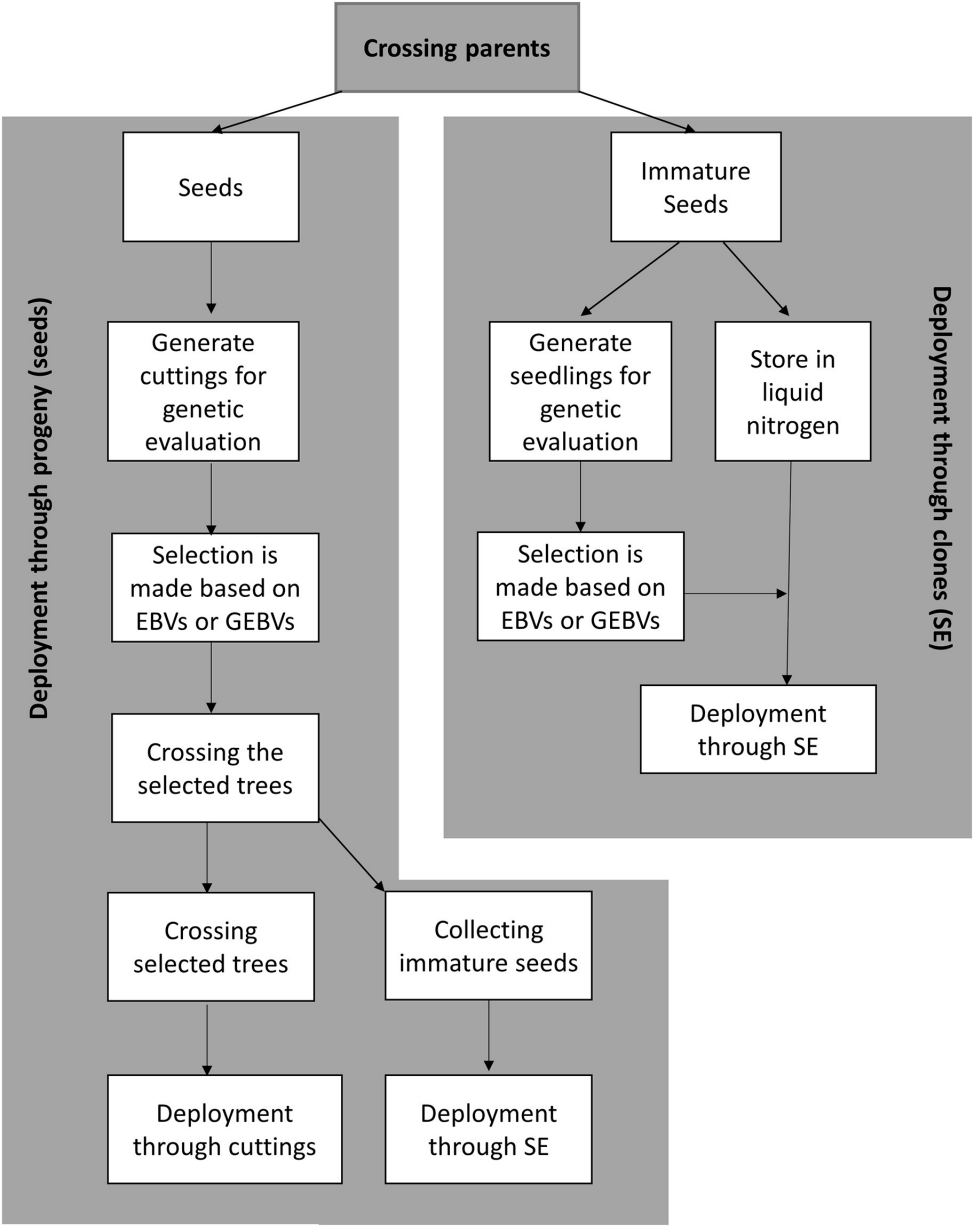
Two deployment options: Deployment of progeny or clones of the selected trees.

Two deployment pathways were simulated to ensure the best selections reach the forest: deploying progeny or deploying clones (Fig 3). The first option is to generate seeds by crossing the selected trees, establishing individual plants as stool beds (mother plants) from which cuttings are taken and then deploying rooted cuttings into the forest with the following steps: graft the selected trees (three years) → cross and generate seeds (three years) → either i) sow seeds or ii) use embryogenesis to generate trees for planting (one additional year) → stool plants (one year) → deployment in the forest. It therefore takes eight years from selection to deployment in the forest for FS. For the forward selection with clonal archive (FSCA) and genomic selection (GS and GSTG) pathways, traditional grafting is not needed, so that only five years from selection to deployment in the forest are required.

The second option is to deploy clones of selected trees, where immature seeds (embryos) of all progeny are collected during step 1 of the breeding cycle as described in Figs 1 and 2. Somatic embryogenesis is then used to generate plants which are subsequently established as stool beds to produce cuttings. (We have assumed all genotypes are able to go through this process). Tissue of all the genotypes tested in the field are preserved in liquid nitrogen (-120°C) while the trees are grown and can be assessed. Once selections are made from field tests, phenotypes or GEBVs, the preserved cell lines of the selected trees are thawed and cuttings are prepared via stool beds in the nursery, for large-scale clonal deployment. Generating and deploying cuttings to the forest takes one year.

There are eight combinations for deployment between the four breeding and selection schemes (FS, GS, FSCA, GSTG) and two generic deployment options (via seed or clones). Table 1 summarizes these selection combinations, their generation intervals, numbers of years needed from crossing the parents to selection and from selection to deployment in the forest.

**Table 1:** Four selection schemes in breeding population, their combinations with two deployment options, generation interval, numbers of years needed from crossing parents to selection and from selection to deployment in the forest.

Selection in breeding population	Material of selected trees to deploy	Deployment abbreviation^§^	Generation interval	Years from crossing to selection	Years from selection to deployment
FS	Progeny	FS_s_	17	14	8
FS	Clone	FS_c_	17	14	1
FSCA	Progeny	FSCA_s_	14	14	5
FSCA	Clone	FSCA_c_	14	14	1
GS	Progeny	GS_s_	9	9	5
GS	Clone	GS_c_	9	7	1
GSTG	Progeny	GSTG_s_	7	7	5
GSTG	Clone	GSTG_c_	7	7	1

^§^ Subscription _s_ denotes deploying progeny (seedlings) of the selected individuals and subscription _c_ denotes deploying clones from tissue culture.

### Simulation

#### Population structure

Three steps were used to simulate a population for conducting forward selection or genomic selection and implemented using the QMSim software [47] based on a forward-in-time process [48]. The objective of these steps was to create initial linkage disequilibrium and establish mutation-drift equilibrium.

The first step created a base population that was named “Historical Generations”. This step consisted of running 500 generations with 300 female parents and 300 male parents. Random mating was used to undertake a random union of gametes. No selections were made in this step. This step had a constant population size of 1000. The second step, called “Extended Generations”, consisted of 10 generations with 100 female and 100 male parents. This step had a constant population size of 500.

After “Extended Generations”, ten “Recent Discrete Generations” were simulated in the third step, by selecting 32 female parents and 32 male parents from the last generation. A circular mating design was implemented and each parent was crossed twice. Fifteen offspring were generated in each family and each offspring had 4 ramets. These parameters used in the recent generations mimicked more closely to a conifer breeding program such as the Elite Population in *P*. *radiata* [29]. Selections were made based on BLUP EBVs, estimated using Henderson’s mixed linear model equations [49] and implemented using ASREML-R [50].

Two quantitative traits with low heritability (h^2^ = 0.2) and with high heritability (h^2^ = 0.5) were simulated. Both traits had additive genetic variance of 300 based on similar genetic variances for diameter-at-breast-height (mm) and wood density (kg/m^3^), respectively. Three-hundred and fifty bi-allelic QTLs were assumed to control the traits. The true breeding value of an individual was calculated as the sum of the individual QTL additive effects. Non-additive genetic effects between ramets of the same genotype were sampled from a normal distribution with mean of zero and 15% additive genetic variance. Phenotypes were simulated as the sum of the true breeding value, non-additive genetic effects and random residuals.

Groups of 800, 1000, 2000 or 3000 randomly selected clones from generations 4 to 8 (in the “Recent Discrete Generations”) were used as the training population. All individuals in generation 10 were used as the prediction population. The prediction equations of SNPs or SNP effects were estimated in the training population. We then tested the power of the prediction equations of SNPs estimated from the training population to predict performance on the prediction population.

#### Simulation of the conifer genome

In this study, one 150 cM chromosome and five SNP panels of 600, 1,200, 2,500, 4,900 and 8,000 SNP markers randomly distributed on the chromosome were simulated. This was equivalent to a SNP density of 7,000, 14,000, 30,000, 60,000 and 90,000 SNPs at the whole genome level (Table 2). The markers were neutral in their effects on the traits. Initial minor allele frequencies of these markers were larger than 0.10. The three hundred and fifty modelled bi-allelic QTLs were randomly distributed. Additive allelic effects of QTLs were randomly sampled from a gamma distribution with a shape parameter of 0.50 and a scale parameter of 26. Markers and QTLs were simulated to have a recurrent mutation rate of 10^−5^ in the Historical Generations and the Extended Generations but no mutations in markers or QTLs were simulated in the Recent Generations.

**Table 2:** Five SNP panels were simulated with 600, 1,200, 2,500, 4,900 and 8,000 SNPs in one chromosome, which was equvalent to 7K, 14K, 30K, 60K and 90K SNPs.

Panel	SNPs simulated	SNPs included in deriving GEBV	Equivalent at whole genome level (assuming 12 chromosomes)
7K	600	576	6,912
14K	1,200	1,168	14,016
30K	2,500	2,358	28,296
60K	4,900	4,766	57,192
90K	8,000	7,149	85,788

#### Simulation of training and prediction populations

Four training population with a size of 800, 1000, 2000 or 3000 clones were randomly chosen from the Recent Generations set (generations 4 to 8) to reflect conifer breeding populations under selection. The prediction population were composed of 960 clones from the 10^th^ generation.

### Genetic evaluation

The EBVs of individual clones were used as input parameters for deriving individual-clone GEBVs, estimated using linear individual tree mixed models [51]:

$$y\;=\;\mu +Z_{a}a+Z_{d}d+e$$ (1)

where ***y*** is a vector of trait phenotypes, ***μ*** is the overall mean, ***a*** is a vector of additive genetic effects, ***d*** is a vector of non-additive genetic effects, ***e*** is a vector of random residuals. ***Z***_***a***_ and ***Z***_***d***_ are incidence matrices relating the additive and non-additive genetic effects to phenotype. It was assumed that $var(a)\;=\;{A\sigma }_{a}^{2},\;var(d)\;=\;{D\sigma }_{d}^{2}\;$, $var(e)\;=\;{I\sigma }_{e}^{2}$ and $var(y)\;=\;{Z_{a}AZ}_{a}^{'}+{Z_{d}DZ}_{d}^{'}+{I\sigma }_{e}^{2}$, where ***A*** is the additive genetic relationship matrix, ***D*** is the non-additive genetic relationship matrix and ***I*** is the identity matrix, $\sigma_{a}^{2}$ is the additive genetic variance, $\sigma_{d}^{2}$ is the non-additive genetic variance and $\sigma_{e}^{2}$ is the residual variance [51].

### GEBV estimation

The allele substitution effect for each SNP was computed using Gensel [52], in which a Bayesian method called BayesC [53, 54] was used for estimating marker effects. BayesC fitted a statistical model assuming a known fraction of SNPs as having zero effects, which in the current analysis was set to be π = 0.95. Additive SNP effects were fitted to every SNP in the model:

$$y_{i}\;=\;\mu +\sum_{j\;=\;1}^{k}X_{ij}a_{j}+e$$ (2)

where ***y***_***i***_ is a vector of EBVs estimated from trait phenotypes and pedigree data, ***X***_***ij***_ is the incidence matrix related to copy number of a given allele of individual *i* at SNP *j*, ***a***_***j***_ is a vector of additive effects of SNP *j*, and ***e*** is a vector of random residuals. The priors for ***a***_***j***_ were a mixture of normal distributions as described by Habier et al. [54]. The priors of all SNP effects have a common variance, which had a scaled inverse chi-square prior with parameters *va* = 4.2 and scale $S_{a}^{2}.\;S_{a}^{2}$ is a univariate student’s *t*-distribution, *t* (0,va,$\;S_{a}^{2}$). The effect of a SNP fitted with probability (1-π) comes from a mixture of multivariate student’s *t*-distributions, *t* (0, *va*, ${IS}_{a}^{2}$). The prior for the residual effects is normally distributed with mean zero and variance $\sigma_{e}^{2}\;$(based on an arbitrary allocation as an initial value). Gibbs sampling was used to sample the posterior distribution of model parameters. SNP effects were estimated by the mean of the sampled values. The total number of Markov Chain Monte Carlo iterations used for estimating posterior means of marker effects and variances was 40,000. The EBV of a simulated trait was used as response variable and the accuracy of the EBV was used as weighting factor. The weight for the *i*th individual clone was estimated according to Garrick et al. [55] as $w_{i}\;=\;\frac{\frac{1-h^{2}}{h^{2}}}{c+\frac{1-r_{i}^{2}}{r_{i}^{2}}}$, where *h*^2^ is the heritability, *c* is the part of the genetic variance not explained by SNPs, and *r*_*i*_ is the accuracy of the EBV of the *i*th clone.

GEBVs ($\hat{g}$) were predicted as the linear combination of the SNP substitution effects as

$$\hat{g}\;=\;X\prime \hat{a}$$ (3)

where ***X*** is the matrix of SNP genotypes for each individual clone in the prediction population and $\hat{a\;}$ is the vector of estimated SNP effects. The accuracy of the GEBVs was estimated as the correlation between the GEBVs and the true simulated breeding value.

### Linkage disequilibrium

The underlying assumption of genomic selection is that haplotypes at some loci are in linkage disequilibrium with QTL alleles that affect the traits that are subject to selection. Linkage disequilibrium was created through the Historical Generations and Extended Generations to mimic level of linkage disequilibrium of a conifer population, based on knowledge of the radiata pine breeding population. Linkage disequilibrium at the beginning of the Recent Generations was measured as *R*^2^, the squared correlation coefficient between pairs of loci:

$$R^{2}\;=\;\frac{D^{2}}{P_{A}P_{a}P_{B}P_{b}}$$ (4)

where *D* is the deviation of the observed frequency of a haplotype from the expected with

$$D\;=\;P_{AB}-P_{A}P_{B},$$ (5)

where *P*_*AB*_, *P*_*a*_, *P*_*A*_, *P*_*B*_ and *P*_*b*_ are observed frequencies of haplotypes *AB* and of alleles *A*, *a*, *B*, *b*, respectively. *R*^2^ is a more informative measure of LD as it measure the overall departure from complete independence between pairwise combinations of polymorphisms [56].

**Fig 4:**
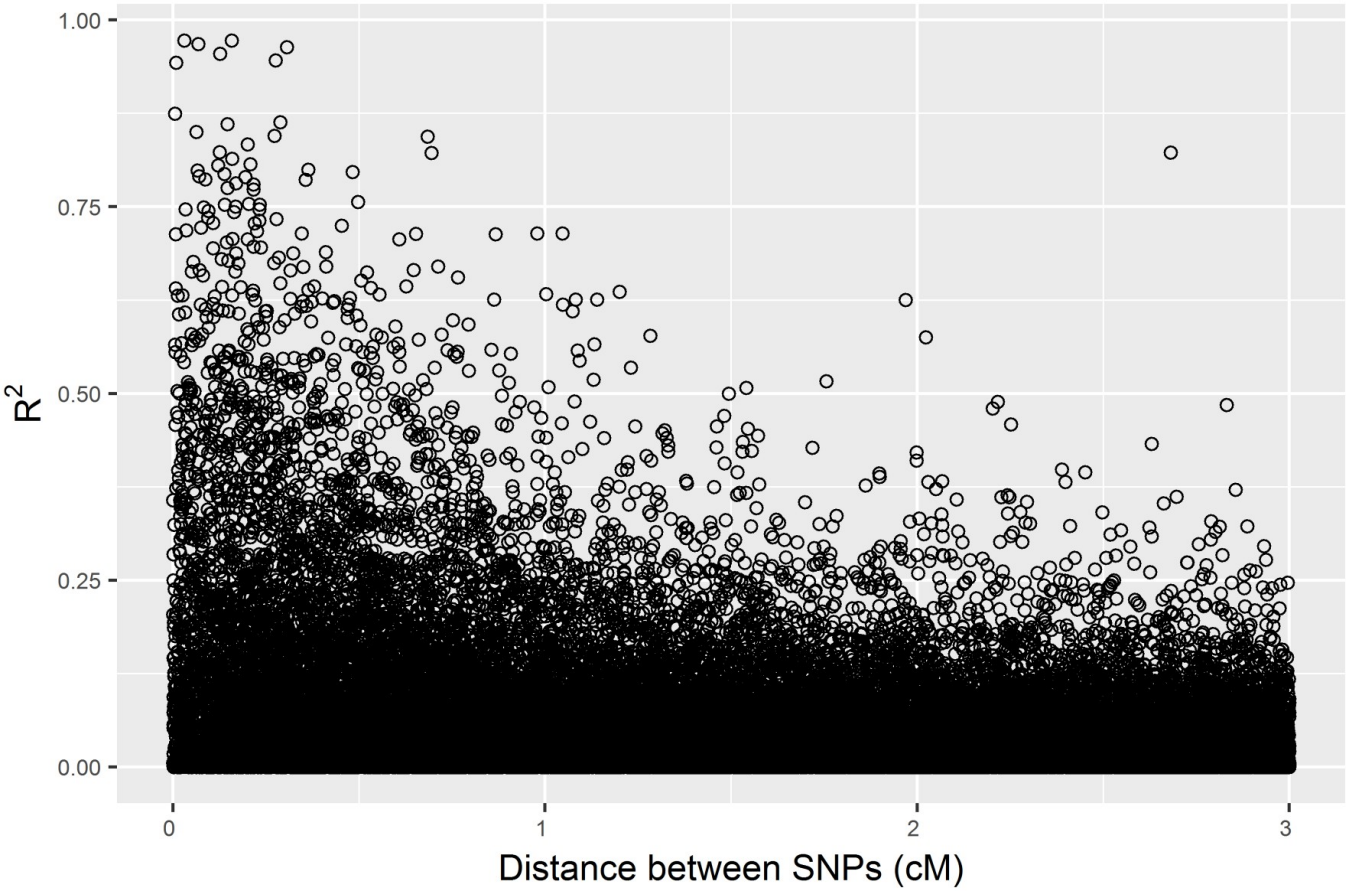
Plot of the squared correlations of allele frequencies (*R*^*2*^) versus distance in cM between closely located SNPs in a panel with 7K SNPs across 150 cM linkage map.

This simulation assumed that the conifer genome has low level of linkage disequilibrium and that linkage disequilibrium rapidly decayed within 1 centimorgan. The average LD was 0.17, 0.20, 0.22, 0.22 and 0.24 for 7K, 14K, 30K, 60K and 90K SNP panels, respectively. Fig 4 shows that the linkage disequilibrium (*R*^2^) rapidly decays with increasing distance between closely located SNPs in the 7K SNP panel.

### Benefit of genomic selection over forward selection

#### Genetic gain in breeding population

In the simulated breeding population, nine clones were selected from 960 clones in the prediction population, which was equivalent to a selection proportion of 1%. Genetic gain was given as the average true breeding value of the selected clones, representing the increase above the population mean of zero. Genetic gain per generation and per year was compared among selections based on their EBVs and GEBVs. Additional genetic gain from selection methods of FSCA, GS and GSTG over FS was calculated in the same manner.

#### Genetic gain in deployment

**Fig 5:**
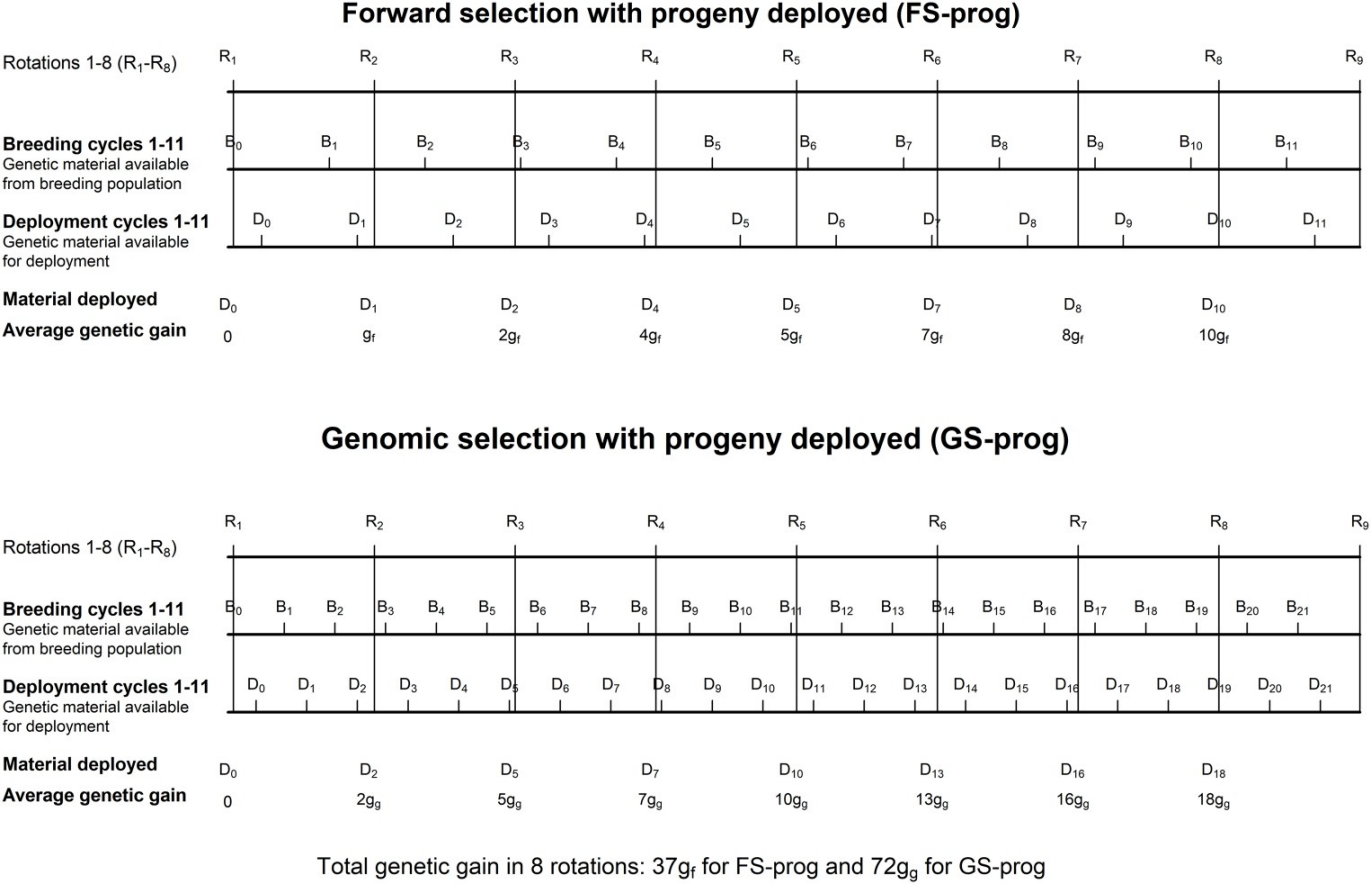
Diagram of calculating total genetic gain from deployment of progeny of the selected individuals for 8 rotations for forward selection (FS_s_) and genomic selection (GS_s_). R_i_ is the start of *i* rotation with 25 years long, B_i_ is the time when selection is made in breeding cycle *i*, D_*i*_ is the time when genetic material is available for deployment in breeding cycle *i*, which is 5 years after selection in breeding cycle *i*. ***g***_***f***_ and ***g***_***g***_ are the genetic gain per generation from FS_s_ and GS_s_, respectively.

Genetic gain from deployment was estimated as the total genetic gain from a plantation of 8 rotations in a hectare of forest. It was assumed that one rotation started immediately after the previous rotation had finished. Each rotation was assumed to be 25 years. The first rotation was assumed to start at the same time that the breeding cycle was started in the breeding population, either for GS or FS. At the start of a new rotation, it was assumed that any deployment could only use the genetic material available from the latest breeding cycle. Genetic merit was the average true breeding value of individual trees selected to be used as parents in the breeding population. The increment of genetic merit in each breeding cycle was assumed to be constant. Genetic merit in breeding cycle *i* was calculated as *g*(*b*_*i*_ − 1), where *g* was the average true breeding value of selected parents and *b*_*i*_ was *i*th breeding cycle. The genetic merit of *i*th rotation was the genetic merit of genetic material that was ready for deployment of the latest breeding cycle. The total genetic gain from a deployment for 8 rotations was calculated as the summation of the average genetic merit of genetic material deployed in 8 rotations. Fig 5 shows an example of how the total genetic gain in the deployment of progeny was calculated for forward selection (FS_s_) and for genomic selection (GS_s_). For instance, the genetic gain in rotation 3 for FS_s_ (2*g*_*f*_) indicates that genetic material from the latest breeding cycle (breeding cycle 2) was deployed. The total genetic gain from a deployment for 8 rotations was estimated as 37*g*_*f*_ for FS_s_ and 72*g*_*g*_ for GS_s_. Additional genetic gain from deployment options of FSCA, GS and GSTG over FS was calculated for two deployment options, deployed as progeny and as clones.

## Results

### Accuracy of GEBVs

The accuracy of GEBVs increased with the increase in the number of individuals in the training population, heritability of the trait of interest, and the density of SNP markers (Table 3). The accuracy of GEBVs increased from 0.21 to 0.51 for a trait with low heritability and from 0.67 to 0.75 for a trait with high heritability, when the training population size increased from 800 to 3000. The accuracy of GEBVs in genomic selection was close to the accuracy of FS for both traits with either low or high heritabilities when the training population size was 3000. The accuracy of GEBVs increased from 0.28 to 0.45 for a trait with low heritability and from 0.51 to 0.70 for a trait with high heritability, when the SNP density increased from 7K to 90K across the simulated chromosome (Table 4). The accuracy of the GEBVs for a trait with a low heritability was much lower than that for a trait with a high heritability for SNPs panels with various numbers of SNPs.

**Table 3:** Accuracies of GEBVs using a 60K SNP panel and a different number of individuals (800, 1,000, 2,000 and 3,000) in the training population for low (h^2^ = 0.2) and high (h^2^ = 0.5) heritability traits, compared with the accuracy of equivalent EBVs.

No. of individuals in the training population	h^2^ = 0.2		h^2^ = 0.5	
	GEBV	EBV	GEBV	EBV
800	0.21	0.52	0.67	0.75
1000	0.43	0.52	0.69	0.75
2000	0.44	0.52	0.73	0.75
3000	0.51	0.52	0.75	0.75

**Table 4:** Accuracies of GEBVs for different numbers of makers in different sizes of SNP panel: 7K, 14K, 30K, 60K or 90K SNPs used in the prediction population for low (h^2^ = 0.2) and high (h^2^ = 0.5) heritabilities, compared with the accuracy of equivalent EBVs. Marker effects were estimated using 1,000 clones in the training population.

SNP density	h^2^ = 0.2		h^2^ = 0.5	
	GEBV	EBV	GEBV	EBV
7K	0.28	0.52	0.51	0.75
14K	0.37	0.52	0.58	0.75
30K	0.40	0.52	0.60	0.75
60K	0.43	0.52	0.69	0.75
90K	0.45	0.52	0.70	0.75

### Genetic gain in the breeding population

Genetic gain was used to evaluate the efficiency of genomic selection when compared with forward selection using phenotypic data and field testing. Genetic gain per generation increased from 4.22% to 16.02% for a trait with low heritability and 18.90% to 24.20% for a trait with high heritability when the number of clones used in the training population increased from 800 to 3000 (Table 5). For a trait with low heritability, the genetic gain per generation from GS was always lower than that from FS across all training population sizes examined. For traits with a high heritability, genetic gain from GS was higher than that from FS when the training population size was 2000 or more. Additional genetic gain was observed in traits with higher heritability.

**Table 5:** Genetic gain per generation obtained in the prediction population from GS with 60K SNP panel for different training population sizes (800, 1,000, 2,000 and 3,000 clones) for traits with low (h^2^ = 0.2) and high (h^2^ = 0.5) heritabilities in the breeding population, compared with genetic gains obtained from FS.

Training population	h^2^ = 0.2		h^2^ = 0.5	
size	GS	FS	GS	FS
800	4.22	19.13	18.90	21.30
1000	10.47	19.13	19.21	21.30
2000	14.23	19.13	21.97	21.30
3000	16.02	19.13	24.20	21.30

Table 6 shows the simulated genetic gain expected in the breeding population per year from FSCA, GS and GSTG relative to the genetic gain achieved from FS. The largest genetic gain was obtained from GSTG. Genetic gain per unit of time in FS was 1.13 for trait with a low heritability and 1.25 for trait with a high heritability. The use of a clonal archive in FS (FSCA) increased gains/year by 21–22%. Genetic gain also increased with the increase of the size of the training population. For the low heritability, when the training population size was 1000, yearly genetic gain from GS was similar to that from forward selection (FS) but lower than the gain from forward selection with a clonal archive (FSCA). When the training population was 2000–3000 clones, genomic selection (GS) led to 40–58% more genetic gain/year than FS. Accelerating coning in genomic selection with top-grafting (GSTG) led to 33%, 80% and 103% additional genetic gain/year over FS for training population sizes of 1000, 2000 and 3000, respectively. For the highly heritable trait, GS resulted in 68–115% additional genetic gain/year and 116–177% more for training population sizes of 800–3000, compared with forward selection (FS).

**Table 6:** Genetic gain per year obtained in the breeding population from genomic selection with (GSTG) or without (GS) top grafting and from forward selection with (FSCA) and without (FS) clonal archive for traits with low (h^2^ = 0.2) or high (h^2^ = 0.5) heritabilities for various training population sizes of 800, 1,000, 2,000 and 3,000. GEBVs were estimated using a 60K SNP panel.

Heritability	Training population size	Genetic gain/year				Benefit over FS		
		FS	FSCA	GS	GSTG	FSCA	GS	GSTG
h^2^ = 0.2	800	1.13	1.37	0.47	0.60	21%	-58%	-47%
	1000	1.13	1.37	1.16	1.50	21%	3%	33%
	2000	1.13	1.37	1.58	2.03	21%	40%	80%
	3000	1.13	1.37	1.78	2.29	21%	58%	103%
h^2^ = 0.5	800	1.25	1.52	2.10	2.7	22%	68%	116%
	1000	1.25	1.52	2.13	2.74	22%	70%	119%
	2000	1.25	1.52	2.44	3.14	22%	95%	151%
	3000	1.25	1.52	2.69	3.46	22%	115%	177%

### Genetic gain in deployment

**Fig 6:**
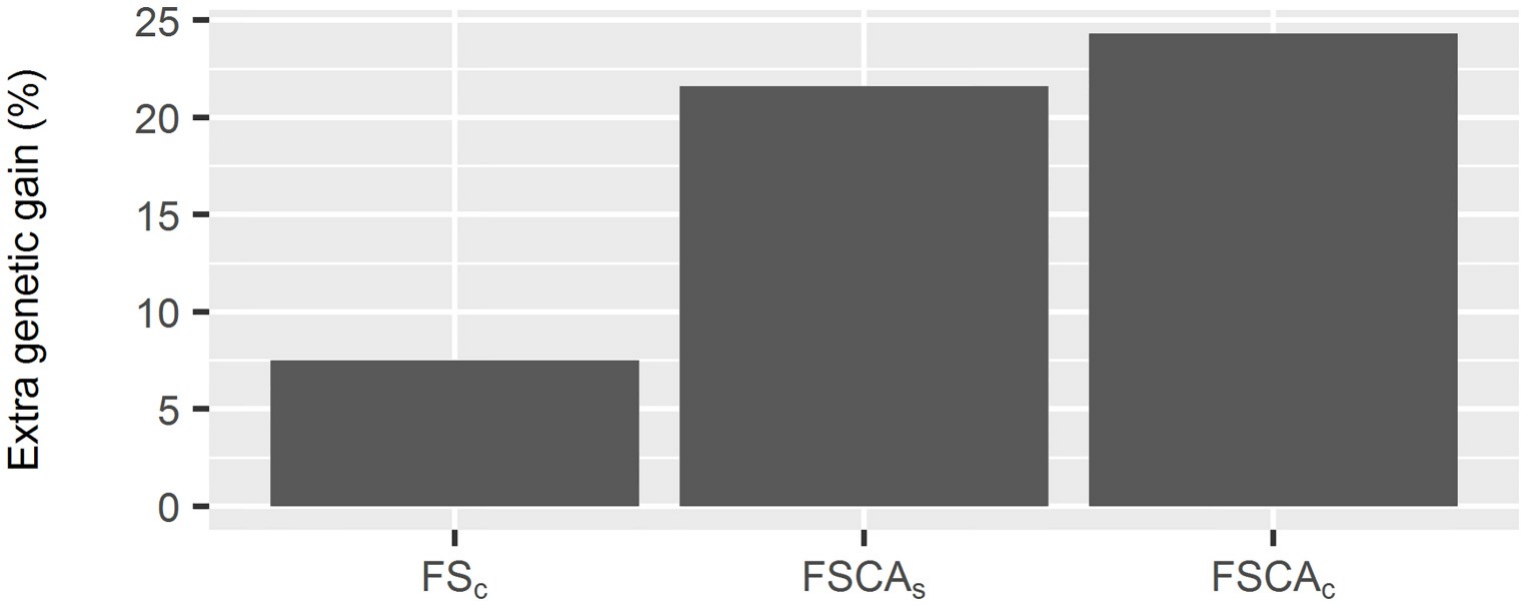
Additional genetic gain per year from FSCA when deployed as seedlings (FSCA_s_), or clones (FSCA_c_), when compared with deployment of selected individuals from FS via seedlings (FS_s_).

Deploying clones through somatic embryogenesis after FS (FS_c_) led to 8.11% additional genetic gain over deploying progeny (seedlings) of the selected individuals (FS_s_) (Fig 6). Use of a clonal archive in combination with forward selection led to considerably higher genetic gain in deployment than forward selection alone. Additional genetic gain available from deploying progeny of the selected individuals through a clonal archive (FSCA_s_) was 21.62%. Deploying individuals via a clonal archive followed by clonal propagation (FSCA_c_) provided an additional 24.32% compared with FS_s_.

Compared with the baseline of deploying progeny of the selected individuals from FS (FS_s_), the additional genetic gains obtained from deploying genetic material selected from GS increased with increasing heritability and training population size (Table 7). Deploying genetic material selected with GS was always superior to deploying genetic material selected from FS except where the training population had 800 individuals and the heritability was low (0.2). Increasing the training population size always increased the additional benefit obtained from deploying genetic material selected from GS over FS. Deploying genetic material selected from GS was inferior to deploying material selected from FS when the heritability was low and the training population size was 800. This suggested that a bigger training population size was needed for a trait with low heritability.

**Table 7:** Additional genetic gains (percentage) across 8 rotations obtained from deploying genetic material selected from GS over a baseline of deploying progeny of the selected individuals from FS (FS_s_) for different deployment options: Deploying progeny (seedlings) of the individuals selected from GS (GS_s_), deploying clones of the individuals selected from GS (GS_c_), deploying progeny of the individuals selected through GSTG (GSTG_s_) or deploying clones of the individuals selected through GSTG (GSTG_c_).

Heritability	Training population size	GS_s_	GS_c_	GSTG_s_	GSTG_c_	GS_c_ vs GS_s_	GSTG_c_ vs GSTG_s_	GSTG_s_ vs GS_s_	GSTG_c_ vs GS_c_
h^2^ = 0.2	800	-57	-56	-45	-42	1	3	12	14
	1000	7	9	38	43	2	5	31	34
	2000	45	49	87	95	4	8	42	46
	3000	63	67	110	120	4	10	47	53
h^2^ = 0.5	800	73	77	123	133	4	10	50	56
	1000	76	80	127	136	4	9	51	56
	2000	101	106	159	170	5	11	58	64
	3000	121	127	186	198	6	12	65	71

With genomic selection (GS and GSTG), deploying clones was always superior to deploying progeny (seedlings) of the selected individuals (Table 7). The additional genetic gain obtained from deploying clones of the selected individuals (GS_c_) was 1–4% and 4–6% higher than that from deploying progeny of the selected individuals (GS_s_) for a trait with low heritability and for a trait with high heritability, respectively. The additional genetic gain obtained from deploying clones of the individuals selected from GSTG (GSTG_c_) was 3–10% and 9–12% higher than that from deploying progeny of the individuals selected from GSTG (GSTG_s_) for a trait with low heritability and high heritability, respectively.

When comparing genetic gains obtained from deploying genetic material selected from GS, the use of top-grafting to accelerate coning remarkably increased genetic gains from deployment (Table 7). Deploying progeny selected from GSTG_s_ led to 12–47% and 50–65% higher additional genetic gain than deploying progeny selected from GS_s_ for a trait with low heritability and high heritability, respectively. Deploying clones selected from GSTG_s_ led to 14–53% and 56–71% higher additional genetic gain than deploying clones selected from GS_s_ for a trait with low heritability and high heritability, respectively.

## Discussion

The objective of this study was to simulate the effectiveness of genomic selection over traditional selection using phenotypes and pedigree information in conifer species. There are several key components to achieve this: increasing the accuracy of estimated breeding values, shortening the generation interval of the breeding population and shortening the time frame from the point of selection to deployment in the forest. The accuracy of genomic breeding values increased with the training population size and the number of markers included in developing genomic breeding values. These accuracies were in the range of those achieved in genomic selection in the literature. The accuracy of GEBVs for growth and wood density was 0.30–0.83 in loblolly pine (*Pinus taeda* L.) [57]; 0.52–0.69 for growth and 0.71–0.79 for wood density in white spruce (*Picea glauca*) [58, 59] and; 0.42–0.65 for wood quality traits and 0.63–0.76 for growth in black spruce (*Picea mariana*) [17]. The simulated accuracy achieved from genomic selection was lower or equivalent to that achieved in forward selection (Tables 3 and 4), which reflected the population size and the number of markers used in most genomic selection projects in conifers [17, 57–59].

While this study focussed on increasing accuracies with increasing marker densities, there are other considerations when designing genomic breeding programmes. This includes designing the genotyping tools to be tailored to the population and traits. In this study higher marker density and higher heritabilities gave higher accuracies of estimated breeding values. Other genomic selection simulations have shown that increasing marker density will better describe the genetics of traits when compared with traditional genetic analysis, for example the recent simulation by [60]. High marker density is the most effective where the heritability is low and where the effective population size is larger. Although this trend is the same for higher heritability traits, marker density is not as important. Of course, this is largely intuitive, but an important point that has not been covered in this study.

The heritability of a trait is an important as it effects the efficiency of genomic selection over traditional selection [10]. It has been assumed that genomic selection is more beneficial where traits have a low heritability [5, 11]. The current results do not support this assumption, as higher additional genetic gain from genomic selection was observed with higher heritabilities over forward selection both in breeding and deployment pathways. It is possible that where heritability is low, more markers or a bigger training population is needed in order to obtain reliable accuracy of GEBVs. Two quantitative traits with h^2^ = 0.2 and h^2^ = 0.5 were simulated. These two traits were representative of the growth trait diameter-at-breast-height (DBH) and wood density. Extrapolating from the current simulation under forward selection, and single-trait selection, we can expect an increase of 1.13 mm per year for DBH and 1.25 kg/m^3^ per year for wood density. Over a 17-year breeding cycle, the expected increases would be 19.13 mm for DBH and 21.3 kg/m^3^ for wood density. With genomic selection, using 2000 individuals in the training population and 60K SNPs for developing GEBVs, we can expect an increase of 1.58 mm per year in DBH and 2.44 kg/m^3^ per year for wood density. After one generation (9-years), this would be equivalent to 14.23 mm for DBH and 21.97 kg/m^3^ for wood density.

This simulation is a generic study for evaluating the efficiency of genomic selection in conifer breeding, assuming very simple situations, such as simulating a single chromosome, no missing phenotypic and genomic data, and discrete recent generations. In reality, wood density has a high heritability and is expensive to measure. It has not been normal, historically, for example to assess wood density for all individuals in a trial. The level of linkage disequilibrium (LD) in a genome is also a key factor that affects the accuracy of genomic selection. This simulation therefore attempted to mimic linkage disequilibrium in conifers using knowledge from radiata pine. Low levels of LD and a rapid decay of LD have been observed in conifer genomes. LD decays about 50% from r^2^≈0.5 to r^2^≈0.25 over 2000 base pair segments in *Pinus taeda* [61] and from r^2^≈0.25 to r^2^≈0.10 over 2000 base pair segments in *Pseudotsuga mensiesii* [62]. The average LD in this simulation was 0.17 to 0.24 for 600 SNPs to 8000 SNPs over one chromosome, which was equivalent to a density of 7K to 90K on the whole radiata pine genome, as a model genome. Chromosome length used in this study was 150cM based on data from previous molecular genetics studies at Scion, which implied 1800 cM in length for the whole radiata pine genome. The possible number of base pairs is over 20 Gb in conifers and 25 Gb in radiata pine [63]. However, due to the low recombination rate in conifers [64], a genetic linkage map might not be larger than other species that have a smaller number of base pairs [65]. However, the genome length used in this simulation is still short compared with genome lengths in some other species. For example, the genome length is 2730 cM in humans [66] and 2300 cM for cattle [67].

Juvenility is generally defined as the period during which a plant cannot be induced to produce cones [68, 69]. Some species of conifers initiate coning only a year or so after being established through grafting or rooted cuttings in a nursery, for example, 10–12 months for *Pinus mugo* [70]. *Pinus* species such as *P*. *banksiana* Lamb., *P*. *virginiana* Mill., and *P*. *contorta* Dougl. var. *contorta* begin coning within 3–5 years of germination [71]. In most forest tree species, the time from seed germination to the onset of coning is about 10 years whereas, in some species, coning does not begin until 25–35 or more years after germination, such as *Fagus sylvatica* L. and *Picea abies* (L.) Karst [72]. Research has been initiated to develop accelerated breeding techniques to reduce the number of years between selection and crossing [73]. The current study showed that top-grafting has the potential to effectively reduce generation interval of conifers and potentially increase genetic gain per unit of time. Examples of using top-grafting as a tool to accelerate coning for conifers include *P*. *taeda* L., where generation interval can be shortened to a minimum of three years and breeding cycle to five years [74–76].

The onset of sexual reproduction will no doubt be a major barrier to shortening the generation interval even further than simulated in this study. The current study assumed that generation interval can be reduced by two years. The time reduced through the use of top-grafting might be different for different conifer species. Coning can, however, be accelerated in conifers using a number of methods which could also be considered. Coning can be accelerated through wide spacing, fertilization and irrigation, the development of full tree crowns and rapid early growth are promoted so that trees reach coning size as soon as possible [77]. Gibberellins, plant hormones that accelerate growth and induce or promote coning of plants, have also been used to accelerate coning in conifers. Gibberellins were used to induce coning from 4–5 years to 3 months for Arizona cypress (*Cupressus arizonica* Greene), from 20–25 years to 12 years for Norway spruce (*Picea abies* (L.) Karst), from 10–20 years to 6 years for Douglas-fir (*Pseudotsuga menziesii*, (Mirb.) Franco) from 20–30 years to 3 years for western hemlock (*Tsuga heterophylla* (Raf.) Sarg.) [78]. Coning of jack pine (*Pinus banksiana* Lamb) can be induced from 5–10 years in naturally regenerated stands under open-grown conditions to 12 months under near optimum growing conditions in the greenhouse and nursery [79, 80]. Time to coning was effectively reduced by application of top-grafting and gibberellins in scots pine (*Pinus sylvestris* (L.)) [81]. Early-coning in conifers can be also achieved through transformation of the gene Coning Locus T (FT), an important coning regulator, which causes plants to cone at a very young age when the plants are quite small [82–84].

One impediment that prevents somatic embryogenesis to be applied commercially in clonal forestry for most of conifers is its high costs [85]. This problem has been largely solved for some conifer species, for example radiata pine. It has been become a reliable and cost-effective commercial clonal forestry development and delivery system in radiata pine [31]. About four million of radiata pine clonal plants generated through embryogenesis have been deployed annually in New Zealand and this number is expected to be expanded substantially. Production costs for somatic embryogenesis clonal forestry is about two times of that for control-pollinated seedlings with a 100% of successful rate per cross (Carson, personal communication, 2018). With the development of this technology, production costs will be reduced in the future for most of conifer species. Fluidic systems are also under development that increase yield of somatic embryos, which should further increase the competitiveness of clonal systems [86].

Therefore, somatic embryogenesis was simulated as a pathway to deploy genetic gain to the forest. There are likely to be at least two ways of using somatic embryogenesis in conifer breeding programmes, outlined in Fig 3. One pathway is described in this study, where immature seeds of every individual to be tested in the breeding programme are collected, cell lines developed and then used to generate plants for testing or genomic evaluation while also being cryopreserved in liquid nitrogen. Once a selection is made, embryos of the selected individuals can be grown for propagation and deployment. A second pathway is to use somatic embryogenesis when deploying progeny of the selected individuals. Immature embryos are collected after crossing selected parents and replicated through tissue culture for mass propagation via stoolbeds. The time frame from selection to deployment for this option in the current study is equivalent to that for rooting of cuttings. The advantage of this somatic embryogenesis deployment option is that a very large number of copies of a clone can be generated.

Due to the high cost of genotyping, other studies have often only genotyped part of a training population using a high SNP density assay. The remainder of the population is genotyped using a low SNP density assay. Imputation is often used to ‘fill in the blanks’ through SNP associations, and predict genotypes to the levels of a high SNP density assay [87]. We have not considered this strategy here. A possible next step in the simulation would be to consider a more complicated situation of high- and low-density SNP assays with imputation to determine the validity of this approach in conifers. Only two scenarios—low and high heritability traits—were considered. Investigation of more heritability options and multiple traits will be beneficial in informing conifer breeding programs how to best optimise the implementation of GS in their populations.

## Conclusions

The accuracy of GEBVs increased with the increase in the number of individuals in the training population, heritability of the interested trait and the density of SNP markers. Accuracy of GEBVs and genetic gain per unit of time for the trait with lower heritability was a key factor determining the minimum training population size, suggesting that 2000 clones in the training population is the minimum size for effective genomic selection for conifers. Deploying clones of the selected individuals always resulted in higher additional genetic gain than deploying progeny. Deploying genetic material selected from genomic selection with top-grafting for early coning resulted in 12–71% additional genetic gain in the deployment than genomic selection without an early coning option. Application of genomic selection to conifer breeding programs, combined with deployment options such as top-grafting and somatic embryogenesis are powerful tools to speed the delivery of genetic gain to the forest.
